# The association of magnetoencephalography high‐frequency oscillations with epilepsy types and a ripple‐based method with source‐level connectivity for mapping epilepsy sources

**DOI:** 10.1111/cns.14115

**Published:** 2023-02-23

**Authors:** Li‐juan Shi, Can‐Cheng Li, Yi‐cong Lin, Cheng‐tao Ding, Yu‐ping Wang, Ji‐cong Zhang

**Affiliations:** ^1^ School of Biological Science and Medical Engineering Beihang University Beijing China; ^2^ Beijing Advanced Innovation Centre for Big Data‐Based Precision Medicine Beihang University Beijing China; ^3^ Beijing Advanced Innovation Centre for Biomedical Engineering Beihang University Beijing China; ^4^ Department of Neurology, Xuanwu Hospital Capital Medical University Beijing China; ^5^ Brain Functional Disease and Neuromodulation of Beijing Key Laboratory Beijing China; ^6^ Hefei Innovation Research Institute, Beihang University Hefei Anhui China

**Keywords:** functional connectivity, MEG, ripple, source location, spatial complexity, tucker decomposition

## Abstract

**Objective:**

To explore the association between high‐frequency oscillations (HFOs) and epilepsy types and to improve the accuracy of source localization.

**Methods:**

Magnetoencephalography (MEG) ripples of 63 drug‐resistant epilepsy patients were detected. Ripple rates, distribution, spatial complexity, and the clustering coefficient of ripple channels were used for the preliminary classification of lateral temporal lobe epilepsy (LTLE), mesial temporal lobe epilepsy (MTLE), and nontemporal lobe epilepsy (NTLE), mainly frontal lobe epilepsy (FLE). Furthermore, the seizure site identification was improved using the Tucker LCMV method and source‐level betweenness centrality.

**Results:**

Ripple rates were significantly higher in MTLE than in LTLE and NTLE (*p* < 0.05). The LTLE and MTLE were mainly distributed in the temporal lobe, followed by the parietal lobe, occipital lobe, and frontal lobe, whereas MTLE ripples were mainly distributed in the frontal lobe, then parietal lobe and occipital lobe. Nevertheless, the NTLE ripples were primarily in the frontal lobe and partially in the occipital lobe (*p* < 0.05). Meanwhile, the spatial complexity of NTLE was significantly higher than that of LTLE and MTLE and was lowest in MTLE (*p* < 0.01). However, an opposite trend was observed for the standardized clustering coefficient compared with spatial complexity (*p* < 0.01). Finally, the tucker algorithm showed a higher percentage of ripples at the surgical site when the betweenness centrality was added (*p* < 0.01).

**Conclusion:**

This study demonstrated that HFO rates, distribution, spatial complexity, and clustering coefficient of ripple channels varied considerably among the three epilepsy types. Additionally, tucker MEG estimation combined with ripple rates based on the source‐level functional connectivity is a promising approach for presurgical epilepsy evaluation.

## Graphical abstract

High‐frequency oscillations rates, distribution, spatial complexity, and clustering coefficient of ripple channel vary among lateral temporal lobe epilepsy (LTLE), mesial temporal lobe epilepsy (MTLE), and nontemporal lobe epilepsy (NTLE, mainly frontal lobe epilepsy, FLE). Additionally, tucker estimation of MEG combined with ripple based on the source‐level functional connectivity proves promising for presurgical epilepsy evaluation.

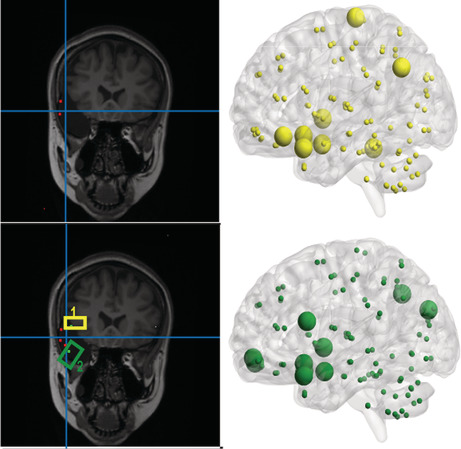

## 1 INTRODUCTION

About a third of epilepsy are clinically refractory to drug therapy and cause significant morbidity.^1^ Surgery is a promising alternative for treating epilepsy.^2^ However, about 20%–50% of epileptic patients undergo experience seizures due to inaccurate resection.^3^ Currently, temporal lobe epilepsy (TLE) accounts for more than 50% of all focal epilepsy cases,^4^, ^5^ while frontal lobe epilepsy (FLE) accounts for approximately 30% of all cases.^6^

Therefore, TLE and FLE are clinically challenging to treat, and effective preoperative classification and evaluation are necessary. There is an urgent need for reliable biomarkers, and more novel and comprehensive evaluation methods to determine the epilepsy type and epileptogenic zone.^7^

High‐frequency oscillations (HFO) (ripple: 80–250 Hz; fast ripple: 250–500 Hz) have been used as a new biomarker of epilepsy for decades,^8^ which is an oscillation with frequencies higher than 80 Hz on electroencephalography (EEG) or magnetoencephalography (MEG).^9^ Among all the electrophysiological presurgical evaluation techniques, such as electroencephalography (EEG), video‐EEG, head MRI, and magnetoencephalography (MEG), EEG is cheaper than most other technologies used in preoperative epilepsy assessment.^10^, ^11^, ^12^ However, EEG has a lower spatial resolution than MEG and is susceptible to noise.^13^ While MEG has increasingly become a valuable noninvasive diagnostic tool for preoperative epilepsy evaluation and has a high temporal and spatial resolution.^14^, ^15^, ^16^

However, compared with EEG, relatively fewer HFO studies in source locations based on MEG have been conducted in the past decades.^17^ So it is a ripe research area. The rates and morphological features of HFO were used to evaluate the seizure status.^18^ Varying HFOs have been observed among different seizure types.^19^ Resection of the HFO‐generating areas is associated with better treatment outcomes than removing the spike‐generating areas. On the other hand, retaining most of the HFO‐generating tissue is associated with poor treatment outcomes.^20^ Compared with spikes, different mechanisms are involved in HFO‐associated seizures.^21^, ^22^ Moreover, the source location accuracy of ripple was higher than spike.^23^, ^24^ The ripples detected from MEG are more specific and sensitive to the activated brain areas than from EEG.^25^ Therefore, there is a need to explore whether various HFO characteristics could be used for the preliminary identification of epilepsy types.

Apart from determining the initial epilepsy type, it is difficult to accurately locate seizure foci for successful epilepsy surgery. Currently, it is also challenging to accurately identify the source location due to the ill‐posed nature of the inverse problem.^26^ Therefore, many techniques have been proposed to solve the inverse problem.^27^, ^28^ Beamforming and the improved linearly constrained minimum variance (LCMV) source location algorithms have been proposed to address the inverse problem.^23^, ^29^, ^30^, ^31^ However, the accuracy of this method needs improvement. To solve the problem, Tucker decomposition, a higher‐order extension of traditional singular value decomposition (SVD) and Principal Component Analysis (PCA), has been used for multichannel signal analysis and showed advantages.^32^, ^33^ Tucker is a method used to estimate the ranks of an N‐order input tensor so that the high‐ and low‐frequency noises can be removed synchronously.^23^, ^34^ While its application in multichannel bioelectric signals is rare.^35^

In addition to considering the new biomarkers and the accuracy of solving the inverse problem, we also innovatively take into account the impaired network activity and connectivity of epilepsy to make a more comprehensive preoperative evaluation.^24^, ^36^, ^37^, ^38^ Analyzing the HFO epileptic network could be used for epilepsy diagnosis.^39^ Most studies on functional connectivity for epilepsy have relied on EEG.^40^, ^41^ To understand the interrelationships among different brain regions, there is a need to explore source‐level functional brain networks. Studies show that the hub state of each region of interest (ROI) in the preoperative evaluation of epilepsy, which relies on betweenness centrality, effectively predicts epilepsy recurrence after surgery.^42^ The maximum accuracy of functional connectivity at the source level relies on the improvement of the source location algorithm because the brain is a complex nonlinear system with nontrivial topological and dynamical properties.^43^ Therefore, the severity of seizures can be predicted by the nonlinear dynamic dimension of MEG.^44^ Mean correlation dimension and Kolmogorov entropy of the nonlinear dynamic system are used to assess the treatment effects of repetitive transcranial magnetic stimulation (rTMS).^45^ Apart from that, chaos theory is used in enhancing the accuracy of the presurgical evaluation of epilepsy based on the spatial and temporal interactions between epileptogenic zones and other parts.^46^

To make noninvasive classification and accurate preoperative evaluation, we propose a multidimensional approach using MEG HFO (ripple) to precisely localize the epileptic focus of MTLE, LTLE, and NTLE (mainly FLE).

The following were the main objectives and contributions of the study:

1. Innovatively, epilepsy types were preliminarily distinguished by ripple rates, ripple distribution, as well as nonlinear dynamic features, and network collectivization.
2. An improved source location algorithm of LCMV through Tucker decomposition, which could remove the redundant information in the MEG signal, was used for more accurate localization of the seizure foci.
3. Source‐level functional connectivity including 90 ROIs based on the improved source location Tucker algorithm was employed to enhance the accuracy of position estimates, which provides great help to the preoperative evaluation of epilepsy.

**FIGURE 1:**
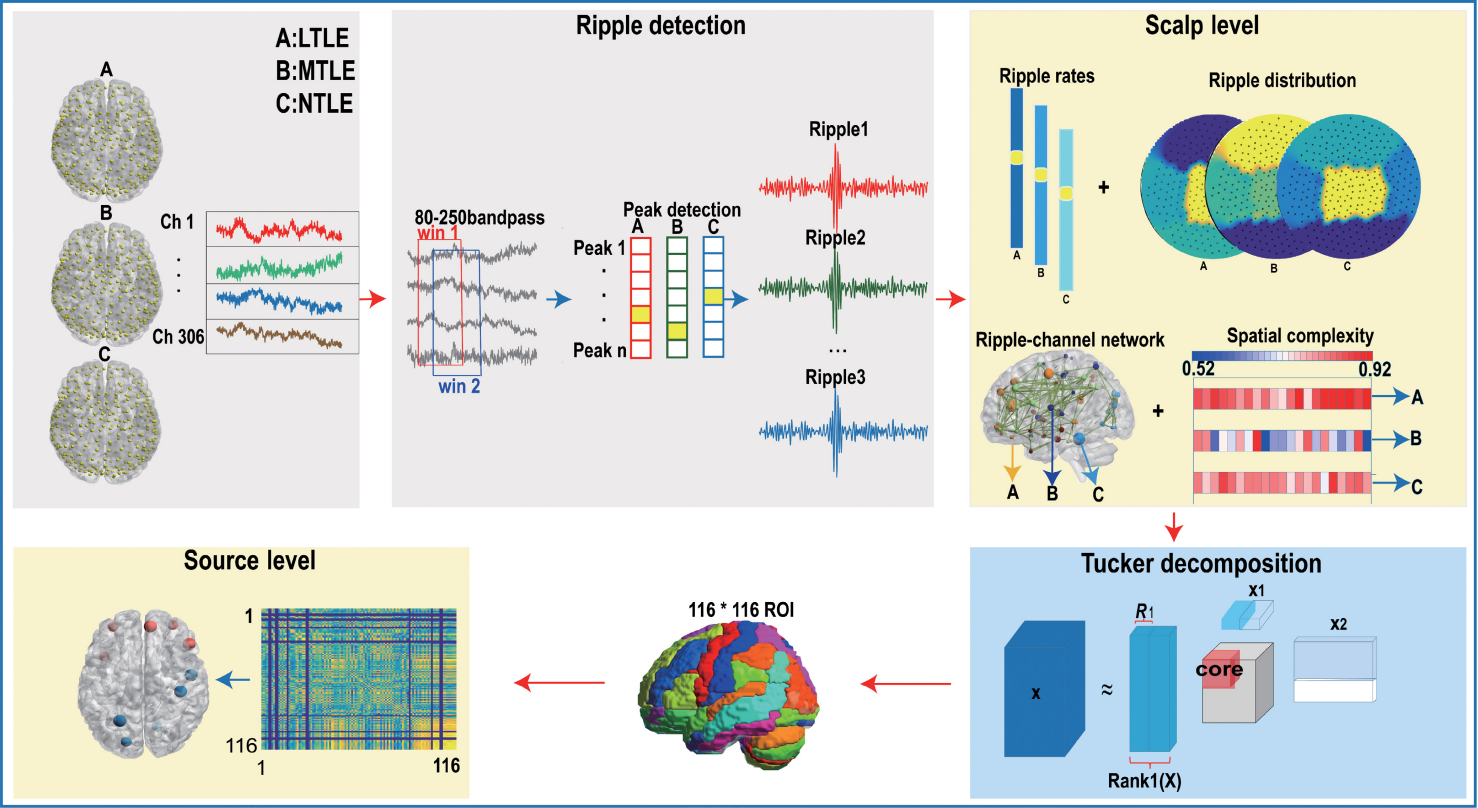
Overview of the preoperative evaluation of lateral temporal lobe epilepsy (LTLE), mesial temporal lobe epilepsy (MTLE), and nontemporal lobe epilepsy (NTLE), including ripple detection and scalp‐level analysis, improved source location algorithm based on Tucker decomposition, and source‐level analysis

The flow diagram of this study is shown in Figure 1.

## 2 METHODS

### 2.1 Subjects

A total of 63 patients with refractory focal epilepsy attending Xuanwu Hospital affiliated with Capital Medical University, Beijing, China, were enrolled in this study. The patients underwent complete standard preoperative assessments (MEG, MRI, video‐EEG, and other assessments). The recruited patients were followed up after 1 year after surgery to assess the clinical outcomes. They are all seizure‐free after surgery over 1 year of surgery. The specific epilepsy subtypes were the following three categories: (1) LTLE (21), (2) MTLE (21), and (3) NTLE (mainly FLE) (21). The clinical details of the patients are shown in Table 1.

**TABLE 1:** The clinical details of the 63 patients.

Patient ID	epilepsy Classification	Sex	Age	Duration (year)	Seizure location by MEG reports	The consistency of MEG reports and resection position
1‐21	LTLE	12F 9M	31 ± 9	10 ± 6	1–5, 8–21: LTL 6: PL 7: PLF	1–5,8–21: consistent 6–7: inconsistent
22‐42	MTLE	9F 12M	33 ± 8	13 ± 8	22–25, 27, 30–38, 37, 42: MTL 26, 39–41: LTL 28–29: FL	22–25, 27, 30–38, 42: consistent 26, ‐28–29, 39–41: inconsistent
43‐63	NTLE	9F 12M	26 ± 9	10 ± 9	43–44, 46, 48‐50, 52–54, 7–59, 61–63: FL 45, 51, 55–56: CA 47: LTL 60: MTL	44–46, 49–50, 52, 61–63: consistent 43, 47–48, 51, 53–60: inconsistent

Abbreviations: CA, central area; F, female; FL, temporal lobe; M, male; PL, parietal lobe; PLF, posterior lateral fissure.

### 2.2 MRI and MEG

Individual MRI: Individual structural MRI image was acquired using a 3.0‐T MRI system equipped with 192 coronal MRI sections (Siemens, Germany). The MRI system was from Xuanwu Hospital of Capital Medical University, Beijing, China. The data were used for constructing volume conduction models.

Magnetoencephalography signals: The MEG signals of 63 subjects were captured by the Neuromag Elekta from Xuanwu Hospital of Capital Medical University at 1000 Hz. The Neuromag Elekta is equipped with 306 channels, and the MEG signals were captured for 1 h. A total of 102 MEG signals were continuously collected. The patients were in a resting state during this process and had their eyes closed.

### 2.3 Preprocessing

The artifacts were removed before ripple detection. Firstly, the line noise was removed using a discrete Fourier transform. Then, electrooculogram and electrocardiograph artifacts were removed through independent component analysis. Lastly, all channels were converted into z‐scores using a semi‐automatic method of rejecting channels containing artifacts, which were all completed in MATLAB. To determine the spatial distribution of ripple, the 306 MEG channels were divided into four brain sections (frontal lobe, temporal lobe, parietal lobe, and occipital lobe).

### 2.4 Automatic detection of ripples

**FIGURE 2:**
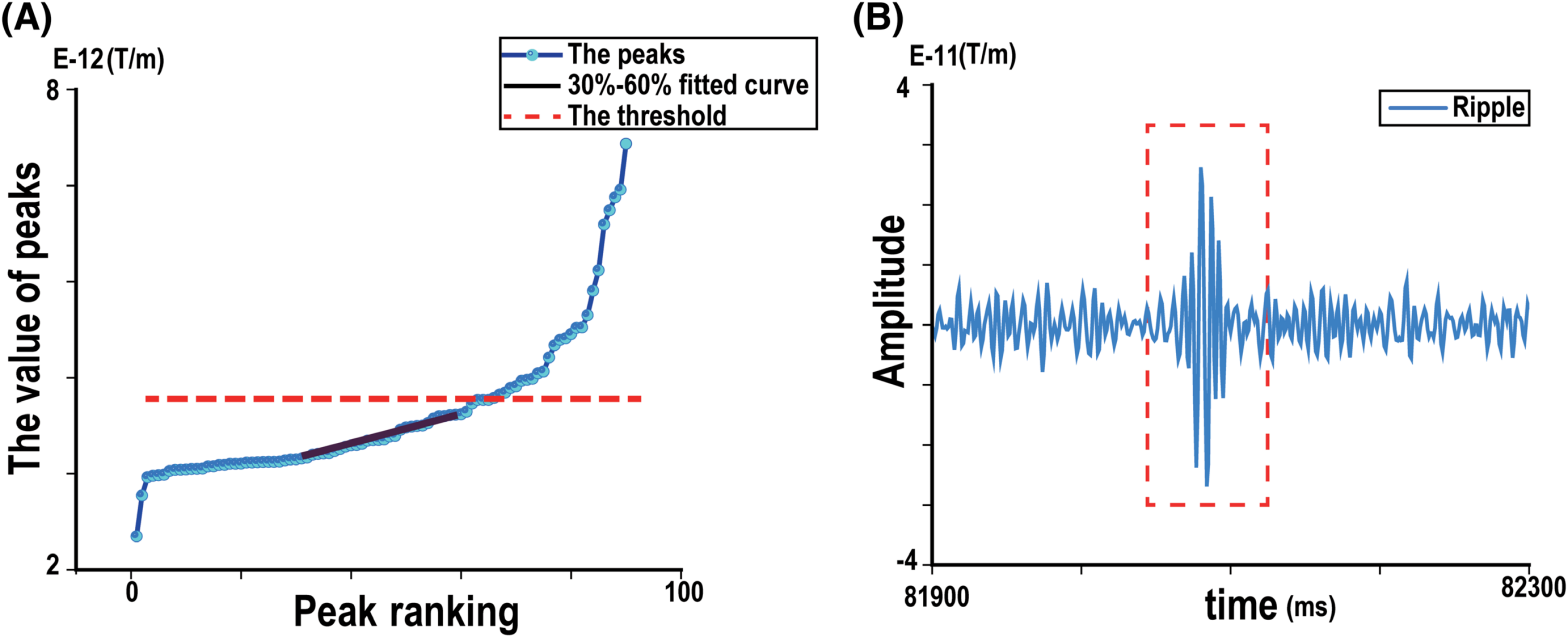
(A) The dark blue line is the fitting straight line of the 30% ~ 60% peaks, the dotted red line is the threshold of the ripples, while the light blue line is the ordered peaks. (B) representative image of magnetoencephalography (MEG) ripple

A sliding window algorithm was used as the basis for the automatic detection algorithm. An improved algorithm based on the root mean square (RMS) of MEG was used after wavelet threshold de‐noising (“db4”) of the MEG signal for 0.6 s^47^ in MATLAB. To construct the peak points distribution curve (PPDC) with 80–250 Hz ripple signal, all signal peaks were detected during the 0.6 s interval and were ranked from highest to lowest. The distribution curve of the 30%~60% peaks was then fitted into a linear model. An amplitude greater than 5% of the fitted amplitude line was used as the boundary of the ripple threshold and baseline for the PPDC (Figure 2A). Furthermore, the dynamic thresholds were created using this improved algorithm. Waveforms with four more continuous peaks higher than the threshold and in accordance with the oscillation trend (rising and falling slowly) were classified as ripples (Figure 2B).

### 2.5 MEG ripple analysis of LTLE, MTLE, and NTLE on scalp level

During the preoperative assessment of epilepsy, we considered three aspects: (1) ripple rate and channel distribution pattern, (2) nonlinear dynamic feature, and (3) scalp networks.

#### 2.5.1 Ripple detection rates and channel distribution pattern

To distinguish the different ripple rates of the three epilepsy types (LTLE, MTLE, and NTLE), the number of ripples in 10 min of the 63 patients was calculated using the MATLAB program.

To compute the spatial distribution of ripple, the 306 MEG channels were divided into four brain sections (frontal lobe, temporal lobe, parietal lobe, and occipital lobe). Except for occipital lobe areas, which cover 72 channels, the rest of the brain areas cover 78 channels. The ripple‐detecting percentage of each brain section was calculated, and then, we assigned the value of all channels in this section to the value of the percentage, so that the 306 channels have a total of four different values. The topographic distribution over the head of 2‐dimensional data representations was plotted using fieldtrip software.

#### 2.5.2 Nonlinear dynamic analysis of channels detected ripples

The spatial complexity represents the mutual independence of brain areas. After the time points of ripples were marked, the spatial complexity was computed for every channel that could detect ripple. The computational time length of spatial complexity is 0.3 s.

Firstly, the principal component analysis of the MEG signal was performed, so that every principal component with eigenvalues were obtained. And then, the eigenvalues were normalized. Subsequently, the spatial complexity was quantified using Shannon entropy as shown in Equation 1:

(1) $$\text{omega}=\exp\left\{-\sum_{i=1}^{m}\lambda_{i}^{'}\log\lambda_{i}^{'}\right\}$$

where $\lambda_{i}^{’}$ is the normalized eigenvalues.

For every patient, the spatial complexity of the channels, which could detect ripples, was averaged. 63 quantified spatial complexity were obtained at last.

#### 2.5.3 Scalp‐network analysis

The relationship between local aggregation at the scalp level and epilepsy types were investigated. Specifically, the coherence between MEG signals from the Fourier spectrum, in which the MEG segment was the ripple window (0.3 s), was calculated using the fieldtrip program. The clustering coefficient was calculated to measure the collectivization of the network. Ultimately, the clustering coefficients of the channels that detected ripples and the other 305 channels were calculated and standardized. The clustering coefficient was computed using Equation (2):

(2) $$C_{k}=\frac{2^{*}e}{n^{*}\left(n-1\right)}$$

where *n* is the number of neighbor nodes.

The standardized clustering coefficient was calculated using Equation (3).

(3) $$C_{s}=\frac{C_{k}-C_{A}}{C_{A}}$$

where *C*_*A*_ is the average clustering coefficient of all channels, and *C*_*k*_ is the clustering coefficient of the channels, which detected ripples.

On the coherence map, the threshold we used is 0.7, each 306 * 306 matrix is converted into an undirected weighted graph by applying the threshold 0.7, and the 306 MEG electrodes represent the vertices. If the coherence value exceeded 0.7, an edge between the corresponding vertices was established, which are neighbors of each other. Finally, we mainly compare the standardized clustering coefficients of the channels that can detect ripple.

### 2.6 Preoperative evaluation on source‐level

The results in section 2.5 were only used to assess the epilepsy type. To better pinpoint the foci of the patients, we used the Tucker decomposition and selected the ripple segment as a time window of interest. Additionally, a source‐level network indicator was calculated for more precise detection.

#### 2.6.1 Source localization algorithm

*Forward problem*: Individual head MRI data were used to construct a realistic‐shaped surface (Single shell) inside the skull for MEG. The anatomical MRI was spatially aligned with head coordinates (Neuromag coordinate system) based on external fiducials markers through rotation, scaling, and translation.

*Inverse problem*: The covariance matrix was computed based on the approximated data of the MEG data to improve the accuracy of the source location. The high‐frequency and low‐frequency noises were removed using Tucker decomposition of the 306 channel MEG signals before the inverse problem was solved with linearly constrained minimum variance beamformers (LCMVs). The Tucker decomposition was derived using Equation (4):

(4) $$\left[GU^{\left(1\right)}U^{\left(2\right)}\cdots U^{\left(N\right)}\right]\overset{\mathrm{def}}{=}G\times_{1}U^{\left(1\right)}\times_{2}U^{\left(2\right)}\cdots \times_{N}U^{\left(N\right)}$$

where G is the core tensor containing the main information, and $U^{\left(i\right)}$ are factor matrices of the original tensor. It is an efficient method to calculate the core tensor and the factor matrices, where singular value decomposition (SVD) replaces the eigenvalue decomposition. All the source location processes are completed on fieldtrip.

The pseudo‐code of the Tucker method is described as follows:
Input: tensor *A* and rank‐*R*.
Output: core tensor and *U*.

Main procedures:

1. Extract number of dimensions and norm of *A*:*N* and norm *A*
2. If numel (*R*) = 1.
  R = R * ones (*N*, 1).
3. *U* = cell (*N*, 1)
4. Initial *U* and fit.
5. Main loop: Iterate until convergence.
6. Return core tensor and *U*.

Then, a new matrix, which includes more useful information of the original signal, is reconstructed by the core tensor and the core tensor.

Lastly, the new matrix was used as input in fieldtrip to perform the LCMV algorithm.

#### 2.6.2 Source‐level network analysis

Brain network analysis and source localization were used to assess whether the results of brain network analysis could improve the accuracy of preoperative assessment. The coherence between source‐level signals was calculated using ripple window‐based source analysis using an equation in MATLAB Equation (5).

(5) $$C_{\mathrm{xy}}\left(ƒ\right)=\frac{{\left|S_{\mathrm{xy}}\left(ƒ\right)\right|}^{2}}{S_{\mathrm{xx}}\left(ƒ\right)S_{\mathrm{yy}}\left(ƒ\right)}$$

where $S_{\mathrm{xy}}\left(ƒ\right)$ is the cross‐spectral power density of two signals, $S_{\mathrm{xx}}\left(ƒ\right)$ and $S_{\mathrm{yy}}\left(ƒ\right)$ represent the auto‐power spectral density.

Moreover, the source activity was interpolated onto the voxels of the Anatomical Automatic Labeling(AAL)template from the Montreal Neurological Institute (MNI). A functional network was constructed using 90 ROIs from the source‐reconstruction parameters over the parcels.

Finally, a network graph measure (betweenness centrality) between the ROIs was computed. The 90 betweenness values were ranked from the largest to the smallest to evaluate the overlap between the betweenness of ROIs and the surgical operation site.

### 2.7 Statistical analysis

Data were analyzed using the Origin software version 2022. The difference in the detection rate, spatial complexity, and clustering coefficient among the groups was evaluated using analysis of variance (ANOVA). Besides, we used Bonferroni multiplicity adjustment for multiple comparisons and paired t‐tests to compare LTLE, MTLE, and NTLE source‐level calculations (LCMV source location, Tucker decomposition source location, and source‐level network analysis). Statistical significance was set at *p* < 0.05.

## 3 RESULTS

### 3.1 Ripple detection rates and channel distribution pattern

To investigate the differences in ripple rates and distribution of 306 channels among LTLE, LTLE, and NTLE, we calculated the number of ripples in 10 minutes and the brain area where the ripple channels were located.

**FIGURE 3:**
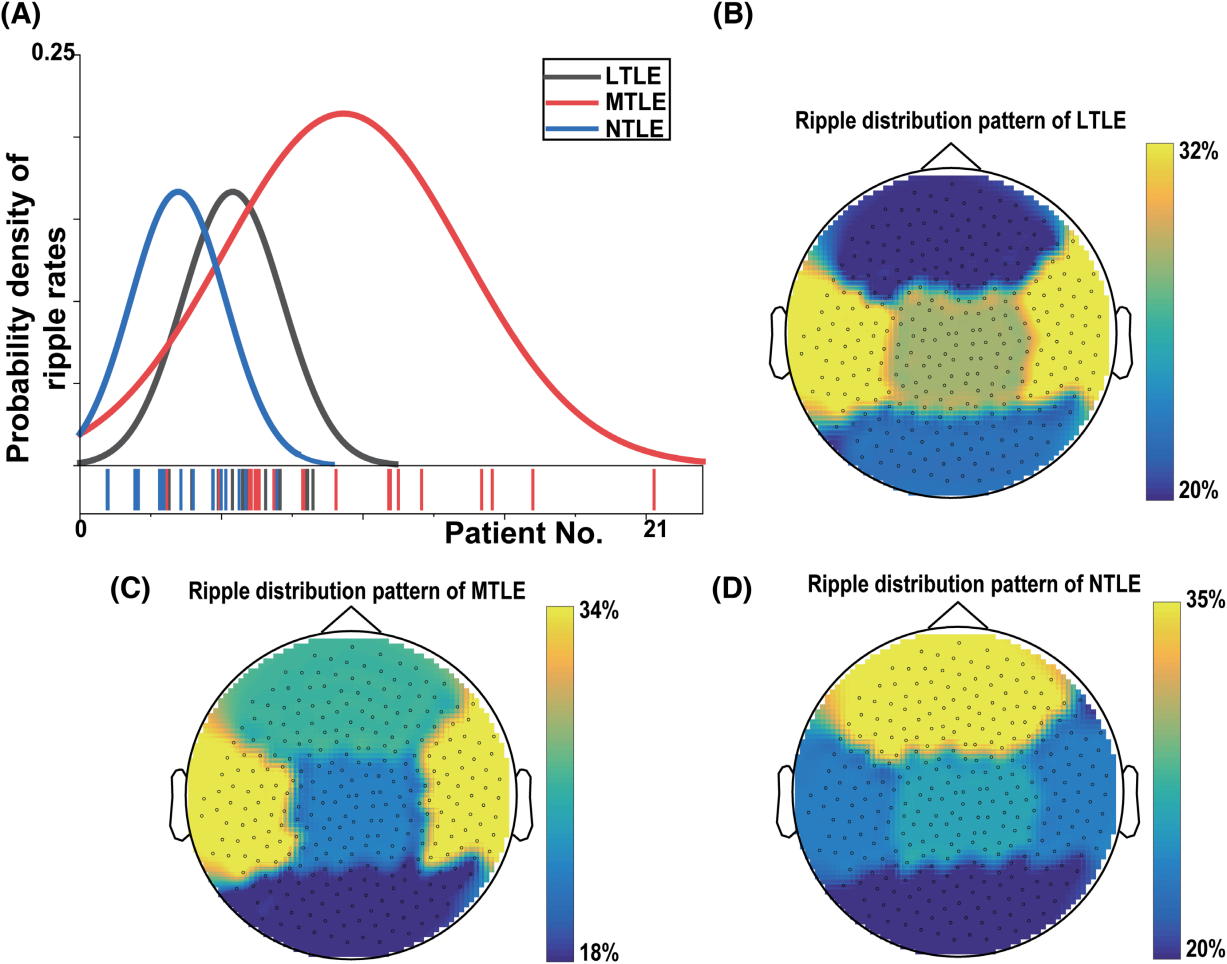
(A) The distribution of ripple detection rates. (B‐D) the distribution of ripples in different brain regions. (B) represents lateral temporal lobe epilepsy (LTLE), (C) represents mesial temporal lobe epilepsy (MTLE), and (D) represents nontemporal lobe epilepsy (NTLE).

Figure 3 A shows the detection rates of the three datasets, which show that ripples of MTLE are higher than those of LTLE and NTLE, and that ripples of LTLE have higher rates than those of NTLE. On average, there were 5.4, 9.3, and 3.5 ripples were found for LTLE, MTLE, and NTLE of 21 cases in 10 minutes. Notably, the rates were significantly different among the three groups (*p* < 0.01).

Ripples of both LTLE and MTLE patients were mainly distributed in the temporal lobe, followed by the parietal lobe and frontal lobe (Figure 3B,C). In NTLE patients, the ripples were mainly distributed in the frontal lobes and least in the occipital lobes. Generally, the firing patterns were significantly different among the epilepsy types (*p* < 0.05). Thus, the ripple rates could be used for the preliminary classification of LTLE, MTLE, and NTLE.

### 3.2 Nonlinear dynamic analysis and scalp‐network analysis

Taking into account the dynamics of ripple channels and the relationship between the three types of ripple channels, the spatial complexity and clustering coefficients were calculated. For nonlinear dynamic analysis, the channels we used were all the channels, which could detect ripples. For scalp‐network analysis, we computed the clustering coefficients for 306 channels, and the clustering coefficient for the ripple channel was standardized using the average clustering coefficient of all 306 channels, which were compared among different epilepsy types. Both the results of nonlinear dynamic analysis and the clustering coefficient were averaged across channels for the same patient.

**FIGURE 4:**
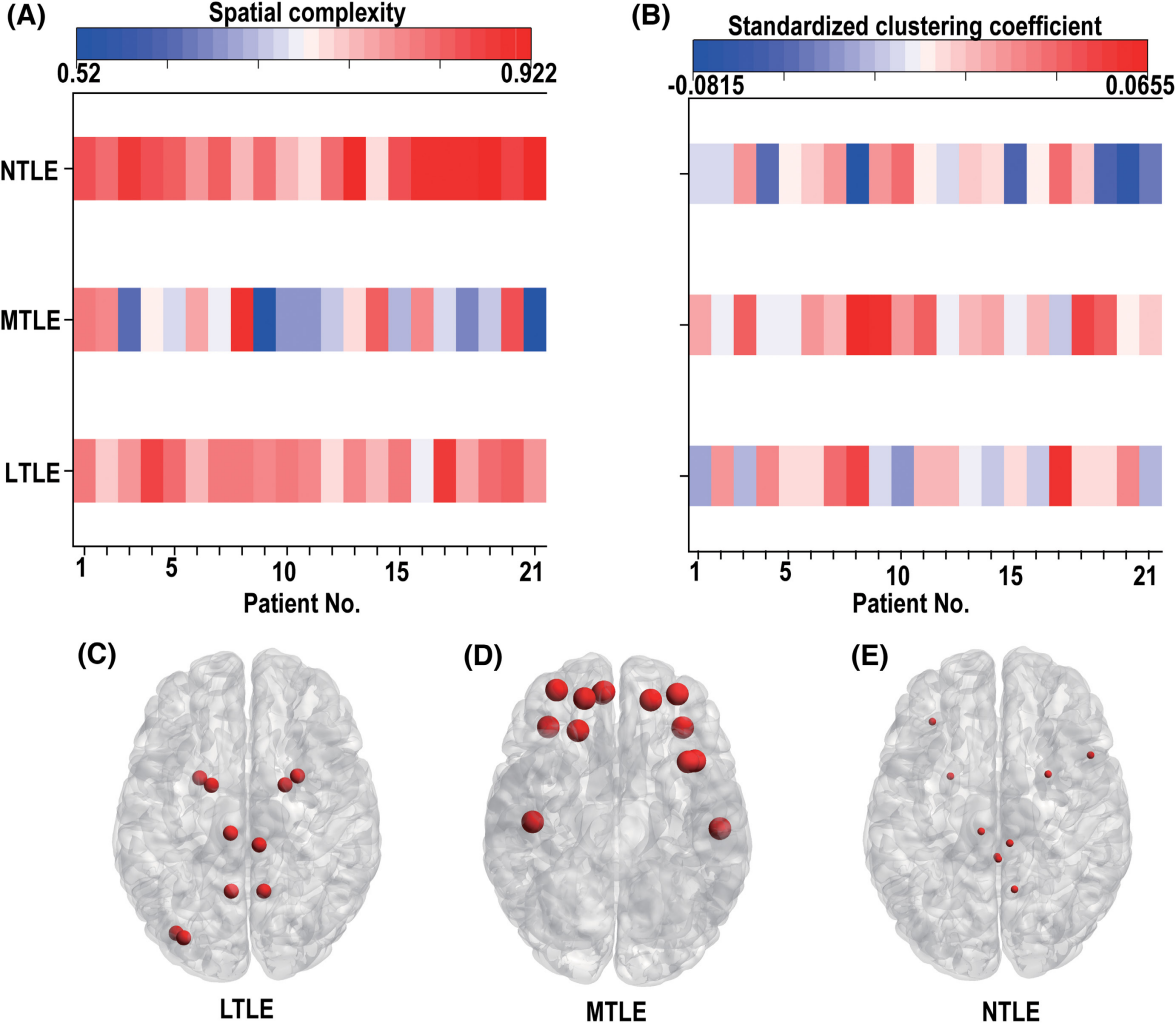
(A) Spatial complexity heat map for the 63 epilepsy patients. The darker colors indicate a higher spatial complexity. (B) A heat map of the standardized clustering coefficient for the 63 epilepsy patients. (C‐E) The node sizes for standardized clustering coefficients of the three epilepsy types (LTLE, MTLE, and NTLE)

As shown in Figure 4A, the spatial complexity was significantly higher in NTLE than in LTLE and MTLE patients, and the spatial complexity was lowest in MTLE patients. The spatial complexity findings were then compared with the number of ripples in 10 min (*p* < 0.01) and were found to be opposite. In contrast with Figure 4A, the opposite results were obtained for the standardized clustering coefficient. The standardized clustering coefficient was highest in MTLE patients. LTLE was second while NTLE came third (Figure 4B; *p* < 0.01). The clustering coefficients are shown in Figure 4C‐E. The node sizes represent the clustering coefficients (C: LTLE, D: MTLE, E: NTLE). The node sizes increased from MTLE to LTLE and NTLE.

### 3.3 Source‐level network analysis based on the Tucker decomposition

**FIGURE 5:**
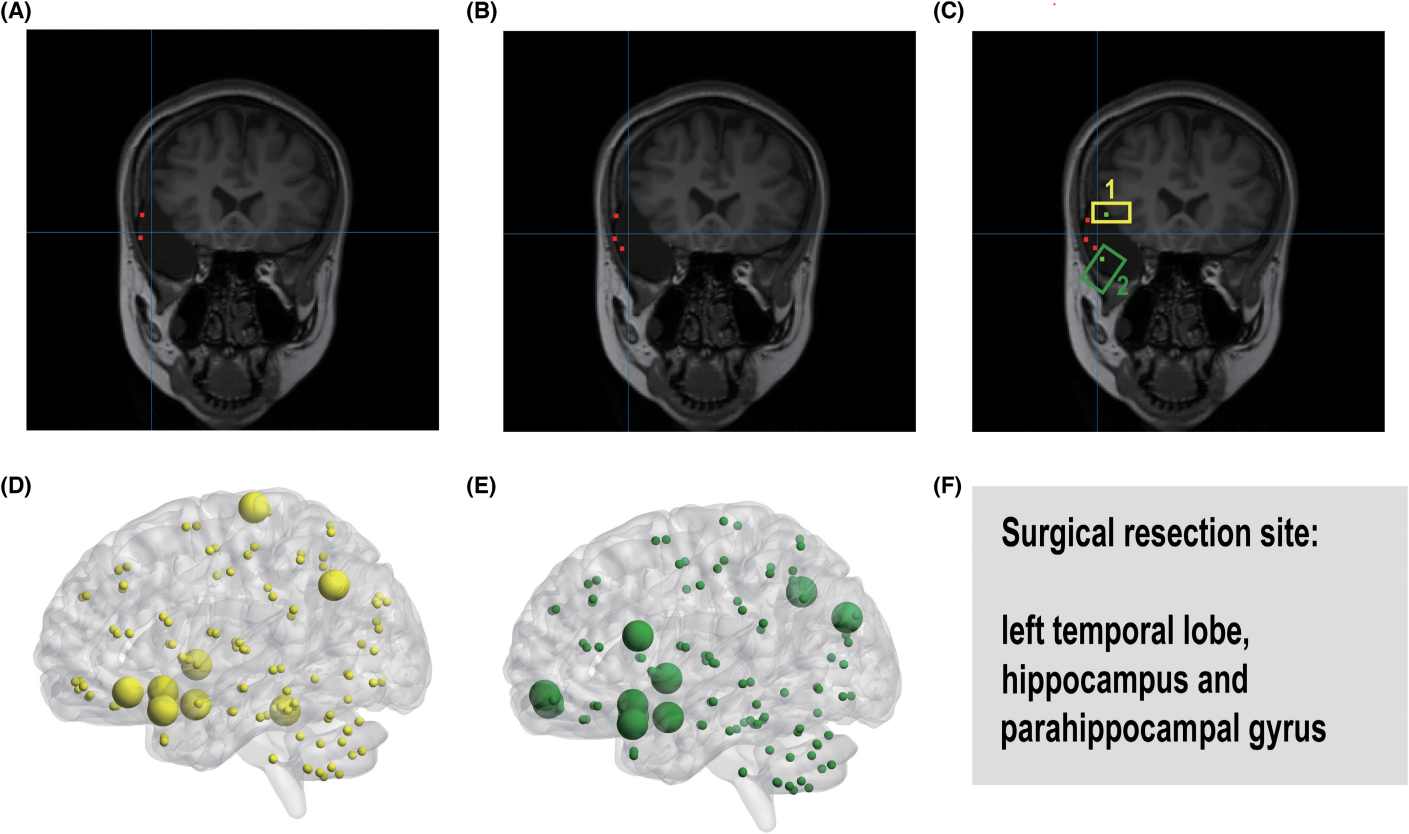
(A) Source imaging results obtained using linearly constrained minimum variance (LCMV), (B) source imaging results obtained by using Tucker LCMV, (C) source imaging results obtained by using Tucker LCMV and source‐level network. The yellow and green points are the two ripples detected in the surgical area. (D) 10 brain areas with the top betweenness of the yellow ripple. (E) 10 brain areas with top betweenness of the green ripple. (F) the surgical resection site

Figure 5A‐C shows a comparison of imaging results using LCMV, the proposed Tucker method, and Tucker with the network. As the result, the ripples located in the surgical resection site increase from left to right. A total of LCMV detected 2 ripples in the surgical area, while Tucker LCMV detected 3 ripples in the same surgical area with Tucker LCMV, and 5 ripples are found in the surgical area when the network analysis was combined, and 2 more ripples were detected. The majority of the top ten betweenness was concentrated in the surgical area using these two ripple windows. Moreover, Figure 5D‐E shows the top 10 betweenness in source level by these 2 ripples: for the yellow ripple 1 and green ripple 2, most areas with top 10 betweenness are in the surgical area: left temporal lobe, hippocampus, and parahippocampal gyrus.

**FIGURE 6:**
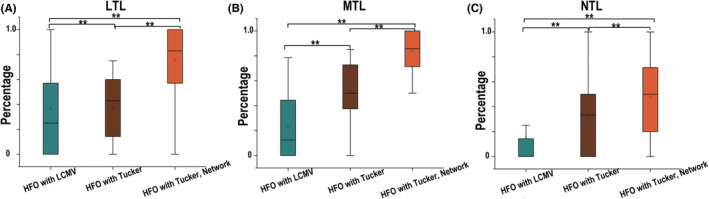
(A‐C) The percentage of ripples at the surgical site identified by linearly constrained minimum variance (LCMV), Tucker LCMV, and Tucker LCMV combined with lateral temporal lobe epilepsy (LTLE), mesial temporal lobe epilepsy (MTLE), and nontemporal lobe epilepsy (NTLE) networks

As shown in Figure 6A‐C, the percentage of ripples at the surgical site was higher than the Tucker algorithm results, and the source location algorithm gave slightly better results than the LCMV algorithm for all three patient categories. Based on the Tucker source location algorithm, the “betweenness centrality” of the 90 ROIs was calculated at the source level. When the “betweenness centrality” was added, the percentage of ripples at the surgical site increased and were higher than those of Tucker decomposition (*p* < 0.01). So functional connectivity analysis effectively supplemented the epilepsy preoperative evaluation with MEG.

## 4 DISCUSSION

In this paper, we investigated the intergroup difference in ripple rates among LTLE, MTLE, and NTLE groups. Specifically, we used a multiangle approach combining ripple rates, ripple distribution, ripple‐channel spatial complexity, and clustering coefficient for initial diagnosis. A new source location algorithm was used to estimate epileptic focus position and network analysis as a supplementary method that improved the location accuracy.

Analysis of the ripple detection rates and distribution revealed that the strength of neural activity varies considerably in different neural areas. For MTLE and LTLE, the neural activity was mostly detected in the temporal lobe, while the second highest rate brain regions are different. Furthermore, the number of NTLEs was higher in the frontal lobes than in the occipital lobes. The ripple‐channel spatial complexity and clustering coefficients showed an opposite trend in LTLE, MTLE, and NTLE. The connectivity strengths of ripple channels were highest in MTLE, moderate in LTLE, and lowest in NTLE, indicating that the higher the spatial complexity, the more independent the neural processes, and the lower the functional connectivity. The localization accuracy of the LCMV method for the ripple window was lower than that of the Tucker LCMV method in the source location. As a consequence, we conclude that Tucker decomposition is more effective at removing redundant noise and keeping the maximum amount of information than LCMV. Functional connectivity at the brain region level revealed that more ripples indicate an area for surgical resection with a higher degree of betweenness centrality. Using noninvasive MEG recordings at the source level to localize the epileptogenic zone is effective in localizing seizures among patients with intractable epilepsy. Analyzing brain networks revealed details of epileptic seizures because an epilepsy network may not be limited to a few regions.

Above all, the proposed framework displays excellent outcomes. These results are consistent with previous studies, in which ripples were demonstrated to contribute to epilepsy pathology by generating and propagating seizures. Thus, ripples are potentially promising epileptogenic biomarkers.^16^

The rates of HFOs with different pathologic substrates were different and were higher in focal cortical dysplasia (FCD), mesial temporal sclerosis, and nodular heterotopia (NH) than in atrophy, polymicrogyria, and tuberous sclerosis.^19^ Thus, HFO rates may represent different pathomechanisms of LTLE, MTLE, and NTLE. Previous studies showed that the highest HFO area was not always located in nodular heterotopias, and some nodular heterotopias were not responsible for seizure onset.^48^ For LTLE, MTLE, and NTLE, the highest HFO area is not around the lesion. HFO networks are dynamic, consistent with epileptogenic network dynamics.^49^ One interesting finding is that the nonlinear dynamics and clustering coefficient, which was performed to examine the relationship between spatial complexity measures and functional connectivity among LTLE, MTLE, and NTLE, showed the opposite trend. So, these four indices were used for the initial classification of the epilepsy types to reduce the burden of epilepsy in the future.

Time window is a critical factor in the accuracy of source localization. It is essential for spikes to be used in clinical evaluation, and the location of the spikes is only used as a reference among the numerous preoperative evaluation methods (Velmurugan et al., 2018). Studies have demonstrated the importance of HFOs in identifying the seizure onset zone (SOZ).^3^, ^7^, ^9^, ^16^, ^17^ Moreover, HFO is superior to spike for preoperative evaluation of an epilepsy type.^23^, ^24^ Therefore, an improved source location method based on the HFO window can effectively improve positioning accuracy. Tucker decomposition based on LCMV provides better source location results than dynamic imaging of coherent sources (DICS), multiple signal classification (MUSIC), and LCMV.^50^ The source location results based on LCMV are more reliable.^23^ Generally, abnormal brain activity is not confined to a single region but spreads to other brain regions through networks.^51^ In this paper, we mapped magnetic field activity onto the source‐level functional modules and found that higher betweenness centrality regions are mostly located in or near the surgical region. Within the focal zone, nodes with higher betweenness centrality are important nodes and they spread out over the entire network. Therefore, epilepsy is a network disease caused by abnormal neocortical connectivity spanning multiple regions.^49^

Overall, these findings further revealed that the pathological mechanisms of the three epilepsy types vary considerably, and the source‐level analysis enhances the identification source location of the three types of epilepsy. Despite these promising results, there are a few areas to address: (1) HFO detection still needs improvement. (2) There might be other indicators that could be used for epilepsy classification. (3) At the source level, whether there were any other functional connectivity metrics that followed certain rules need further investigation. In future work, further investigations will be done to identify epilepsy types and provide a deeper understanding of their pathological mechanisms. In addition, new methods for accurate source localization are needed.

## 5 CONCLUSION

In this study, we investigated the differences in ripple parameters, including ripple rates, ripple distribution, ripple‐channel nonlinear dynamics, and functional connectivity among LTLE, MTLE, and NTLE. The results showed significant differences in these parameters among the three epilepsy subtypes. Moreover, a modified algorithm called Tucker LCMV was used to improve source localization accuracy using a rippled window based on the source‐level network. The proposed method supplemented the source‐level network can be an excellent tool for MEG source localization during the preoperative evaluation of epilepsy. In summary, this study demonstrates that epilepsy types have specific ripple features, and the HFO findings are critical in presurgical assessments.

## AUTHOR CONTRIBUTIONS

Li‐juan Shi conceived the experiments. Li‐juan Shi conducted the experiments and analyzed the data. Can‐cheng Li provided analysis methods. Li‐juan Shi wrote the paper. Yi‐cong Lin and Yu‐ping Wang collected clinical cases and provided clinical guidance. Cheng‐tao Ding provided great help in image modification and language polishing. Ji‐cong Zhang offered a few general observations and broader recommendations, as well as provided funding and support.

## FUNDING INFORMATION

We thank the Beijing Natural Science Foundation (Grant Number: Z200024), the University Synergy Innovation Program of Anhui Province, the Hefei Innovation Research Institute, and the Beihang University (Grant Number: GXXT‐2019‐044).

## CONFLICT OF INTEREST STATEMENT

None of the authors have any financial disclosure or conflicts of interest.

## Data Availability

The data and software code that support the findings of this study are available from the corresponding author upon reasonable request.
